# Phenotypic analyses of rice *lse2* and *lse3* mutants that exhibit hyperaccumulation of starch in the leaf blades

**DOI:** 10.1186/s12284-014-0032-3

**Published:** 2014-12-21

**Authors:** Chenggang Liang, Tatsuro Hirose, Masaki Okamura, Rei Tanimoto, Akio Miyao, Hirohiko Hirochika, Tomio Terao, Tian Li, Ryu Ohsugi, Naohiro Aoki

**Affiliations:** Graduate School of Agricultural and Life Sciences, The University of Tokyo, Tokyo, 113-8657 Japan; NARO Agricultural Research Center, Joetsu, 943-0193 Niigata, Japan; National Institute of Agrobiological Sciences, Tsukuba, 305-8602 Ibaraki, Japan; Key Laboratory of Crop Ecophysiology and Farming System in Southwest China, Ministry of P.C. China, College of Agronomy, Sichuan Agricultural University, Chengdu, 611130 Sichuan, China

**Keywords:** Leaf blade, Oryza sativa, Phloem loading, Photoassimilate partitioning, Starch excess phenotype, Sucrose

## Abstract

**Background:**

To identify genes that potentially regulate the accumulation, mobilization, and transport of photoassimilates in rice (*Oryza sativa* L.) leaves, we recently screened a mutant collection of rice by iodine staining to visualize leaf starch contents. From this screening, we isolated a rice mutant that exhibits hyperaccumulation of starch in leaves and designated it as the Leaf Starch Excess 1 (LSE1) mutant. Here, we report two other rice LSE mutants, LSE2 and LSE3.

**Results:**

Unlike *lse1* plants, *lse2* and *lse3* plants displayed retarded growth; *lse2* showed an extremely dwarf phenotype and rarely survived in paddy fields; *lse3* showed inhibited growth with pale green leaf blades, low tiller numbers, reduced height, and low grain yield. In *lse2* and *lse3* plants, the mature source leaves contained larger amounts of starch and sucrose than those in wild-type and *lse1* plants. Furthermore, microscopic observations of leaf transverse sections indicated that hyperaccumulation of starch in chloroplasts of mesophyll and bundle sheath cells occurred in *lse2* and *lse3* plants, while that in vascular cells was noticeable only in *lse3* leaves.

**Conclusions:**

The distinct phenotypes of these three LSE mutants suggest that the LSE2 and LSE3 mutations occur because of disruption of novel genes that might be involved in the path of sucrose transport from mesophyll cells to phloem sieve elements in rice leaves, the mechanism for which has not yet been elucidated.

**Electronic supplementary material:**

The online version of this article (doi:10.1186/s12284-014-0032-3) contains supplementary material, which is available to authorized users.

## Background

In plants, appropriate carbon partitioning within or between tissues is important for growth, development, and reproduction. Photosynthetically assimilated carbon is converted principally into carbohydrates such as cellulose, starch, sucrose, and hexoses. While cellulose forms the plant’s structure, starch and soluble sugars often accumulate within tissues as non-structural carbohydrates (NSCs) or move from source to sink tissues as translocating sugars, of which sucrose is the most universal among plant species (for recent reviews, see Stitt and Zeeman [2012]; Ruan [2014]). Thus, genetic modification of carbohydrate partitioning has long been proposed in crop breeding (Braun et al. [2014]).

Leaf starch is the most widespread and abundant storage carbohydrate, and is synthesized during the day and hydrolyzed to soluble sugars at night for export from leaves to sites of growth (Perez et al. [1971]; Lloyd et al. [2005]; Smith et al. [2005]; Smith [2012]; Stitt and Zeeman [2012]). Mutants lacking function in genes that are involved in these metabolic processes often display ‘starch excess (sex)’ phenotypes that accumulate excess quantities of starch in the leaves. Since these phenotypes can be easily screened by iodine staining of mature leaves, the genes responsible for *sex* mutations have been extensively studied to explore the molecular mechanisms of carbohydrate partitioning in leaves. By identifying and characterizing *sex* genes, the starch degradation pathway in leaves has been well established in *Arabidopsis thaliana* (L.) Heynh. (for recent reviews, see Zeeman et al. [2010]; Stitt and Zeeman [2012]). However, the starch degradation pathway may vary among plant species. For example, disproportionating enzyme 2, which catalyzes the production of glucose, is located in the cytosol in *A. thaliana* (Chia et al. [2004]) but functions in the chloroplasts in *Solanum tuberosum* L. (potato; Lloyd et al. [2004]). The relevance of maltose transport from chloroplast to cytosol in potato remains unclear (Niittylä et al. [2004]; Lloyd et al. [2005]). Meanwhile, despite the fact that *Oryza sativa* L. (rice) is a model grass species and a major crop that feeds more than half of the global population, the molecular mechanism of starch degradation in the rice leaf has not been elucidated. We recently reported a *sex* mutant by an iodine staining-based screening of a rice mutant collection (Hirose et al. [2013]). Seedlings of this mutant accumulated excess starch in the leaf blades, and the mutant was designated Leaf Starch Excess 1 (LSE1). The LSE1 mutation was determined to be caused by the disruption of a gene encoding α-glucan, water dikinase, *OsGWD1* (Os06g0498400; RAP_DB; http://rapdb.dna.affrc.go.jp/). Despite hyperaccumulation of starch in the leaf blade, the rice *lse1* mutation appeared to have no significant effect on vegetative growth, in contrast to the *sex1*/*gwd1* of *A. thaliana* (Caspar et al. [1991]; Yu et al. [2001]; Ritte et al. [2002]) and the *gwd1* of *Lotus japonicus* (Regel) K. Larsen (Vriet et al. [2010]).

Starch-excess phenomena can also be caused by inhibition of photoassimilate export from leaves. For example, cold-girdling of the stem induces the starch-excess phenomenon by impairing photoassimilate transport (e.g., Krapp et al. [1993]; Slewinski et al. [2009]). In *A. thaliana* and *Zea mays* L. (maize), disruption of genes for a sucrose transporter (SUT) involved in apoplastic phloem loading was found to cause the LSE phenotype (Gottwald et al. [2000]; Slewinski et al. [2009]). In addition to knockout mutants of phloem-loading SUTs, maize mutants including *sed1/sxd1* (Russin et al. [1996]), *tdy1* (Braun et al. [2006]), *tyd2* (Baker and Braun [2008]), and *psc1* (Slewinski and Braun [2010]), were reported to show the LSE phenotype accompanied by inhibition of photoassimilate export from leaves. Although rice is closely related to maize, the molecular mechanism for phloem loading of photoassimilates in rice leaves has not been elucidated. A rice SUT, OsSUT1, was reported to localize in the phloem (Matsukura et al. [2000]; Scofield et al. [2007]). However, antisense suppression of *OsSUT1* did not induce clear symptoms of blocked phloem loading in source leaves (Ishimaru et al. [2001]; Scofield et al. [2002]). More recently, using an *OsSUT1* knockout mutant with the anther culture technique, Eom et al. ([2012]) excluded the possibility that OsSUT1 plays a major role in phloem loading. Another rice SUT, OsSUT2, was reported to play an essential role in photoassimilate export from source leaf blades in rice, while this SUT localizes to the tonoplast membrane of leaf mesophyll cells (Eom et al. [2011]). Thus, the path of photoassimilate transport from mesophyll tissue to phloem sieve elements in rice leaves remains unclear (for recent reviews, see Eom et al. [2012]; Braun et al. [2014]).

Here, we report two other rice LSE mutants, designated as LSE2 and LSE3. To investigate the roles of the target genes conferring the LSE2 and LSE3 mutations, putative homozygously mutated lines were established for LSE2 and LSE3; carbohydrate contents and plant growth in *lse2* and *lse3* were compared with *lse1* and wild-type (WT) plants. The *lse2* plants displayed severely dwarf phenotype, probably caused by the hyperaccumulation of starch and sucrose in leaf blades and sheaths. In contrast, *lse3* plants displayed growth impairment that was intermediate between that of *lse1* and *lse2* plants, and exhibited hyperaccumulation of starch and elevated sugar levels only in the leaf blades. These distinct phenotypes, in combination with different patterns of starch accumulation in leaf tissue, suggest that the roles of *LSE2* and *LSE3* in leaf carbohydrate partitioning differ from that of *LSE1*, and that *LSE2* and *LSE3* are more likely to be involved in sucrose export from leaves than in starch degradation.

## Results

### Establishment of *lse2* and *lse3* mutants of rice

To compare inheritance of the LSE2 and LSE3 phenotype to that of LSE1, segregation analysis was conducted for each LSE mutant line using the M_3_ generation from stain-negative M_2_ plants (Table 1). Similar to LSE1, the stain-positive phenotype in LSE2 was segregated at a ratio of 0.22, suggesting that the phenotype was due to a recessive mutation of a single gene. The segregation ratio of the stain-positive phenotype in LSE3 was 0.16; however, the LSE3 phenotype appeared to be caused by a recessive mutation of a single gene, because the ratio was significantly higher than the theoretical segregation ratio of 0.0625 that assumes two genes are responsible for the phenotype. This hypothesis was supported by the fact that a putative homozygously mutated line, in which all M_3_ individuals showed the stain-positive phenotype, was established for the LSE3 as well as for the LSE2 mutation (see Methods for details). Therefore, we decided to use the putative homozygous lines as the pure lines for the LSE2 and LSE3 mutations to characterize *lse2* and *lse3* plants, respectively. Meanwhile, similar to the LSE1 mutant line, Southern blot analysis revealed that neither the LSE2 nor the LSE3 phenotype was tagged by the *Tos17* retrotransposon, indicating that these phenotypes were caused by some other mutations (data not shown).

**Table 1:** **Segregation ratios of LSE1, LSE2, and LSE3 mutant lines**

LSE mutation^a^	Number of stain-positive plants	Number of stain-negative plants	Segregation ratio of stain-positive plants	***P***-Value^b^
LSE1	25	84	0.23	0.69
LSE2	36	129	0.22	0.71
LSE3	10	68	0.13	0.01

^a^Heterozygously mutated lines were used for this analysis; 109, 165, and 78 seedlings of LSE1, LSE2, and LSE3, respectively, were subjected to iodine staining.

^b^Probability calculated by χ^2^-test when the hypothetical segregation ratio of stain-positive plants is 0.25.

### Phenotypic comparisons of *lse1*, *lse2*, and *lse3* plants grown under controlled glasshouse conditions

**Figure 1:**
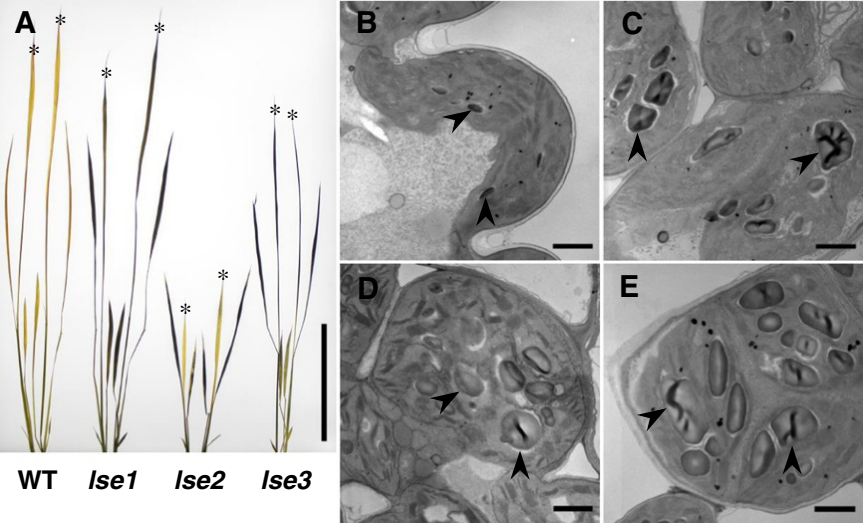
**Starch-excess phenotypes of rice** ***lse*** **mutants at the fifth leaf stage.** Rice seedlings were sampled in the morning and subjected to iodine staining **(A)** or to microscopic observation **(B–E)**. **A**, typical results of iodine staining in WT, *lse1*, *lse2*, and *lse3* seedlings. Note that the youngest fifth leaf blades were still elongating very slowly and were not stained in *lse2*. Asterisks indicate the fifth leaf blade of rice seedlings. Bar = 10 cm. **B–E**, representative TEM images of transverse sections of fully-developed leaf blades of WT (B), *lse1* **(C)**, *lse2* **(D)**, and *lse3* **(E)**. The fourth leaf blades were used for *lse2*; fifth leaf blades were used for the others. Arrowheads indicate starch granules in mesophyll chloroplasts. Scale bars = 1 μm.

**Figure 2:**
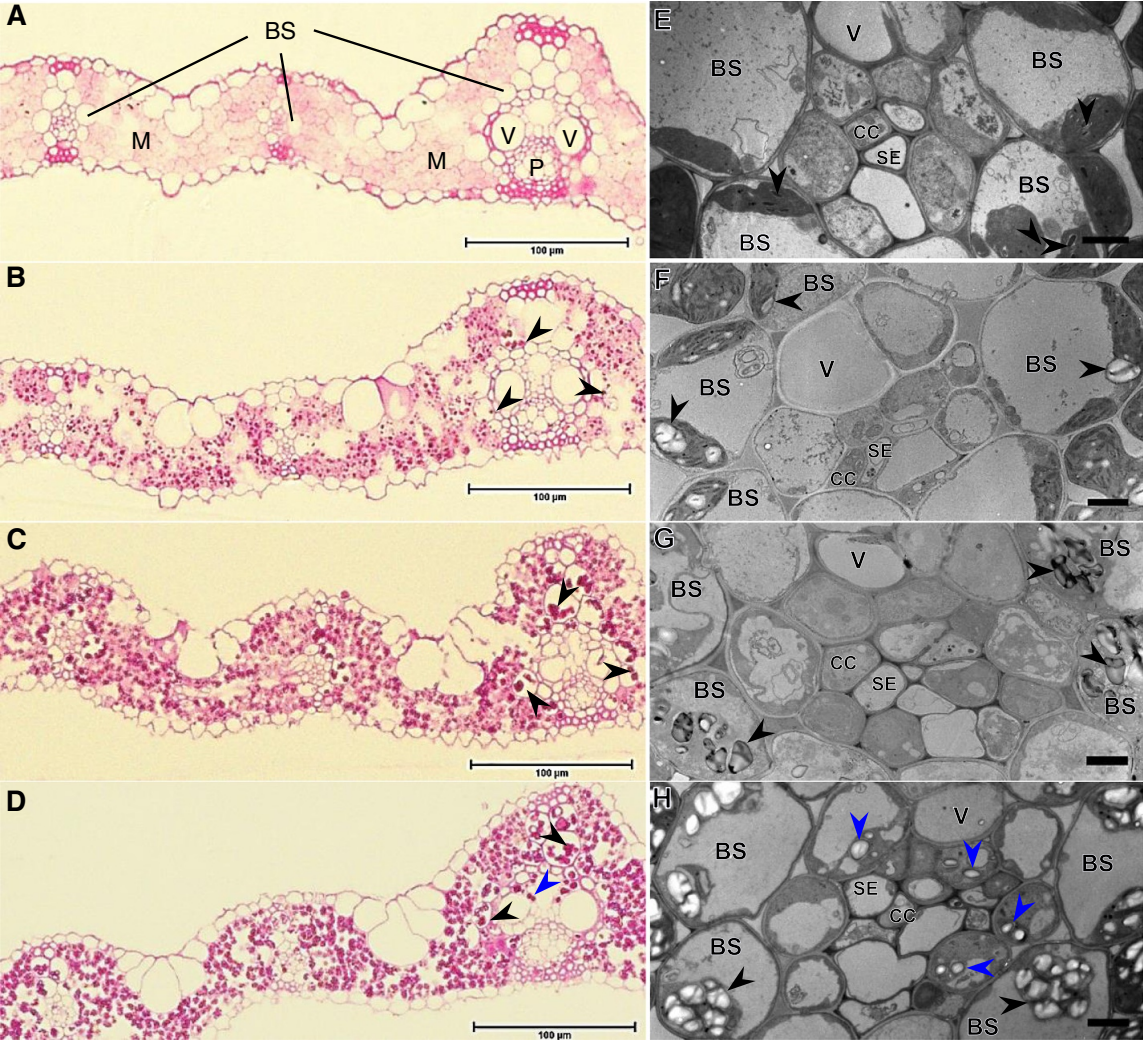
**Microscopic observations of transverse sections of fully developed leaf blades of seedlings.** The same tissue samples as shown in Figure 1B–E were used. **A–D**, light micrographs of periodic acid–Schiff-stained semithin sections. Dark red or purple dots showing accumulation of polysaccharides can be seen in *lse1* **(B)**, *lse2* **(C)**, and *lse3* **(D)**, and not in WT **(A)**. Polysaccharide accumulation was pronounced in mesophyll cells (M) and was also evident in bundle sheath cells surrounding vascular bundles (black arrowheads). Blue arrowheads indicate polysaccharide accumulation within the large vascular bundle in *lse3* **(D)**. Scale bars = 100 μm. E–H, TEM of small or medium vascular bundles in wild type **(E)**, *lse1* **(F)**, *lse2* **(G)**, and *lse3* **(H)**. Black arrowheads indicate starch granules in bundle sheath chloroplasts; blue arrowheads Indicate starch granules in vascular parenchyma cells. Scale bars = 2 μm. BS, bundle sheath cell; CC, companion cell; M, mesophyll; P, phloem; SE, sieve element; V, vessel element.

To characterize *lse2* and *lse3* plants, we first compared them to *lse1* and WT ‘Nipponbare’ plants at the seedling stage. Figure 1A shows representative results of iodine staining of *lse1*, *lse2*, *lse3*, and WT seedlings. The *lse1* plants appeared to grow similarly to the WT, consistent with previous observations (Hirose et al. [2013]). Compared to *lse1* and WT, both *lse2* and *lse3* exhibited impaired growth: *lse2* plants were very short and the height of *lse3* was intermediate between *lse1* and *lse2*. When seedlings were sampled just after the end of the 12-h dark period, almost no starch-accumulation signals were observed in any leaf blades of the WT. On the other hand, the fully matured leaf blades of *lse* seedlings were deeply stained with iodine. This result was consistent with TEM images of the transverse sections of leaves; many extraordinarily large starch granules were clearly observed in mesophyll chloroplasts of the three *lse* mutants (Figure 1C–E), while only a few small starch granules was observed in WT leaves (Figure 1B). To compare starch accumulation patterns in leaves, the transverse sections were stained with PAS solution to detect polysaccharides under light microscopy (Figure 2A–D). Again, deep staining was observed in all *lse* plants (Figure 2B–D) but not in WT plants (Figure 2A), showing starch hyperaccumulation or other polysaccharides in *lse* leaves. While deeply stained polysaccharides were observed not only in mesophyll cells, clear signals of polysaccharide accumulation were also detected in bundle sheath cells in leaves of the three *lse* mutants. The consistent presence of large starch granules in bundle sheath chloroplasts of *lse* leaves was observed under TEM, showing more prominent accumulation of starch compared to bundle sheath chloroplasts of WT leaves (Figure 2E–H). Furthermore, PAS-stained polysaccharides were often observed in the vascular tissues of *lse3* leaves (Figure 2D), in vascular parenchyma cells (Kaneko et al. [1980]; Chonan et al. [1981]; Botha [2013]). The presence of starch granules within the vascular tissue was evident only in *lse3* (Figure 2H).

**Figure 3:**
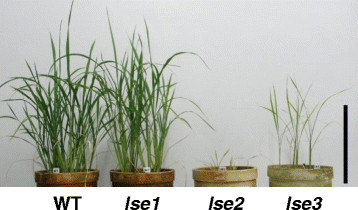
**Two-month-old plants grown in pots under glasshouse conditions.** Scale bar = 20 cm.

Figure 3 shows 2-month-old WT, *lse1*, *lse2*, and *lse3* plants grown in pots. As reported previously, the vegetative growth of *lse1* was similar in appearance to the WT, with a slight decrease in tiller number (Hirose et al. [2013]). Compared with WT and *lse1* plants, *lse2* displayed an extremely dwarf phenotype with yellowish leaves and no tiller, and rarely survived thereafter (Figure 3). The *lse3* plants again exhibited a growth phenotype that was intermediate between that of *lse1* and *lse2* plants, with pale green leaves and no tiller.

**Figure 4:**
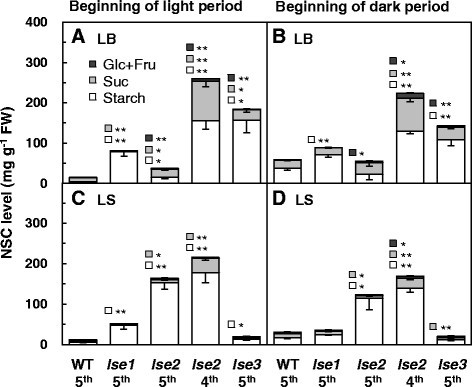
**Comparison of concentrations of NSCs between WT and** ***lse*** **mutants.** Leaf blades (LB) and sheaths (LS) of fifth-leaf-stage seedlings were sampled at the beginning of the light period **(A,C)** and at the beginning of the dark period **(B,D)**. The fifth leaf blades and sheaths (5^th^) were used for determination of starch, sucrose (Suc), glucose (Glc), and fructose (Fru). The fourth leaves (4^th^) were also used in *lse2*. Values represent the averages of four plants ± SE. Differences between the WT and each *lse* mutant were analyzed by Student’s *t*-test. Significant differences are indicated by asterisks (*P ≤ 0.05; **P ≤ 0.01).

To further characterize the accumulation profiles of NSCs in the *lse* plants, the concentrations of starch, sucrose, glucose, and fructose were determined in leaf blades and sheaths at the fifth-leaf stage (Figure 4), from samples collected at the beginning of the light (early morning) and the night (early evening) periods. Based on the iodine-staining patterns shown in Figure 1A, the leaf blades and sheaths of the fifth leaf of WT, *lse1*, *lse2*, and *lse3* plants, and of the fourth leaf of *lse2* plants were selected for analysis. In WT plants, as is common in source leaves of many plant species including rice, the NSC levels were lower in the morning and higher in the evening (Figure 4A,B; the data of statistical analysis are not shown). Levels of starch and soluble sugars were very low in the morning because of nocturnal mobilization of NSCs stored in the leaves, and were high in the evening because of diurnal accumulation of excess photoassimilates in the form of NSCs. In comparison with WT leaves, none of the *lse* leaves showed clear day-night changes in NSC levels, suggesting that these leaves maintained extraordinarily high levels of NSCs (especially starch) throughout the day. Consistent with our previous findings (Hirose et al. [2013]), starch levels in leaf blades of *lse1* were significantly higher than that of WT in both morning and evening (Figure 4A,B), while soluble sugar levels in leaf blades did not differ statistically between *lse1* and WT. As shown in Figure 1A, the fifth leaf blades of *lse2* seedlings were still elongating and scarcely stained with iodine even when sampled in the early morning, while the fully-matured fourth leaf blades were deeply stained. This observation was quantitatively confirmed by the determination of NSC levels; no remarkable differences in starch level were observed between the fifth leaf blades of *lse2* and WT, and starch levels in the fourth leaf blade of *lse2* were much higher than those in the fifth leaf blade of WT, both in the morning and evening (Figure 4A,B). Soluble sugar (especially sucrose) levels in the mature leaf blades of *lse2* were significantly higher than those of WT regardless of the sampling time. Starch and sugar levels in the leaf blades of *lse3* were higher than those of the WT regardless of sampling time.

The leaf sheaths of grass species are considered to function as temporal storage tissues for accumulating excess photoassimilates, and as conducting tissues that connect source leaf blades with terminal sink tissues such as roots and developing leaves (for recent review see Slewinski [2012]). In comparison with the WT, leaf sheaths of *lse1* and *les3* contained significantly larger quantities of starch only in tissues sampled in the morning, and those of *lse2* accumulated larger amounts of starch regardless of leaf age or sampling time (Figure 4C,D). Soluble sugar levels in leaf sheaths were similar between *lse* mutants and WT, or were lower in the mutants, except for the fourth leaf sheath of *lse2*, in which sucrose levels were significantly higher than those of WT in both morning and evening.

### Phenotypic comparisons of *lse1*, *lse2*, and *lse3* plants grown under field conditions

We also compared *lse2* and *lse3* plants to *lse1* and WT plants in terms of growth and productivity under field conditions, and obtained comparable results in two different years. Four-week-old seedlings were transplanted into a paddy field and grown in plant communities. The *lse2* seedlings rarely survived under full-sun field conditions, requiring heavily shaded environments (e.g., surrounding one *lse2* seedling with WT seedlings; data not shown) to persist. Therefore, we decided to use only *lse3* for the phenotypic comparisons to *lse1* and WT. First, we examined whether the phenotypic differences observed under glasshouse conditions were maintained in field-grown adult plants at the heading stage. Compared to *lse1* and WT plants, the heading day was delayed in *lse3* plants by more than one week (data not shown), and the plant length of *lse3* was significantly shorter (Table 2). These phenotypic characteristics were consistent with the retardation and impaired growth observed in *lse3* (and *lse2*) at the seedling and vegetative growth stages (Figures 1A and 3).

**Table 2:** **Characteristics of rice plant growth and yield components**

	Plant length^a^(cm)	Panicles per plant	Spikelets per panicle	Percentage of filled grains (%)	Average grain weight (mg)	Yield per plant (g)
WT	95.6 ± 0.9	10.8 ± 0.3	103.1 ± 5.9	76.5 ± 2.4	25.5 ± 0.4	22.0 ± 1.9
*lse1*	92.2 ± 1.1*	7.8 ± 0.5**	84.0 ± 3.9*	70.2 ± 2.7	25.1 ± 0.3	11.4 ± 0.5**
*lse3*	78.6 ± 1.1**	7.8 ± 0.5**	47.8 ± 2.2**	70.5 ± 3.5	24.2 ± 0.2**	6.4 ± 0.7**

^**a**^Plant length was measured at the heading stage and the yield components were measured after harvest.

Values represent the means ± SE (n ≥ 4). Differences between the WT and each *lse* mutant were analyzed by Student’s t-test. Significant differences are indicated by asterisks (*P ≤ 0.05; **P ≤ 0.01).

**Figure 5:**
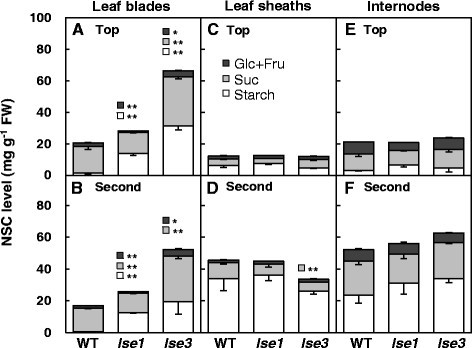
**Comparison of concentrations of NSCs between WT and** ***lse*** **mutants grown under field conditions.** Leaf blades, sheaths, and internodes were sampled between 9:00 and 10:00 AM at the heading stage. The flag (top) leaf blades **(A)** and sheath **(C)**, the second-top leaf blades **(B)** and sheaths **(D)**, the top **(E)** and second-top **(F)** internodes were used for determination of starch, sucrose (Suc), glucose (Glc), and fructose (Fru). Values represent the averages of four plants ± SE. Differences between the WT and each *lse* mutant were analyzed by Student’s *t*-test. Significant differences are indicated by asterisks (*P ≤ 0.05; **P ≤ 0.01).

Next, NSC contents in leaf blades, sheaths, and internodes were compared at the heading stage (Figure 5). In leaf blades, starch levels were significantly higher in *lse1* and *lse3* than in WT, showing the LSE phenotype in both the flag (top) and second-top leaf (Figure 5A,B). Starch levels in leaf sheaths and internodes were similar between *lse1* and WT regardless of the position (Figure 5C–F). Compared to the WT, starch levels in *lse3* plants tended to be lower in leaf sheaths and higher in internodes. Similar to the leaf blades of seedlings collected at the beginning of the light period (Figure 4B), soluble sugar (especially sucrose) levels were significantly elevated in leaf blades of *lse3* plants compared with those of WT and *lse1* plants (Figure 5A,B). In leaf sheaths and internodes, no significant increases in soluble sugar levels were observed in the two *lse* mutants compared with WT plants (Figure 5C–F). Finally, we measured the yield components at harvest (approximately 6 weeks after heading) (Table 2). As reported previously, the grain yield per plant was significantly decreased in *lse1* compared with the WT because of a decrease in all four components: number of panicles per plant, number of spikelets per panicle, percentage of filled grains, and average grain weight (Hirose et al. [2013]). The grain yield of *lse3* was also approximately 70% lower than that of the WT because of considerable reductions in all the components except for percentage of filled grains (Table 2). It should be noted that the shaded *lse2* plants were very short (< 50 cm) and showed little grain yield (approximately 5% of WT) with no tillers, a small number of spikelets per panicle, a very low percentage of filled grains, and very small grain size.

## Discussion

We previously reported screening of a rice mutant collection by iodine staining of seedlings harvested in the early morning (Hirose et al. [2013]), and established a single-recessive rice mutant (*lse1*) that accumulates excess starch. In that paper, the responsible gene, *LSE1*, was identified as encoding an α-glucan, water dikinase involved in starch degradation (*OsGDW1*; Os06g0498400; RAP_DB). Using similar procedures, we obtained two other rice LSE mutants, *lse2* and *lse3*. Whereas hyperaccumulation of starch appeared to occur in chloroplasts in all three LSE mutants (Figure 1), *lse2* and *lse3* showed phenotypes that were distinct from *lse1*. Unlike *lse1* plants, *lse2* and *lse3* plants showed markedly impaired growth (Figures 1 and 3) and accumulated excess starch and sucrose in mature leaf blades (Figure 4). Segregation analysis suggested that the LSE2 phenotype was caused by a recessive mutation in a single gene, while the segregation ratio for the LSE3 phenotype implied non-Mendelian inheritance (Table 1). Although the reasons for the abnormal segregation ratio in LSE3 (0.16) remain to be clarified, it can be assumed that the phenotype is not a result of recessive mutations in two or more genes. Preliminary data from our ongoing gene-mapping work suggest that the LSE3 mutation is located in a single locus (data not shown). Collectively, either *lse2* or *lse3* would be a result of disruption of a single gene distinct from *LSE1* (located on chromosome 6), although neither *LSE2* nor *LSE3* has been identified.

To date, single-gene disruption mutants that accumulate excess starch in source leaves have been reported in *A. thaliana*, *L. japonicus*, *Solanum lycopersicum* L. (tomato), maize, and rice (Additional file 1: Table S1, and references therein). Starch excess in leaves is often observed when inhibition of starch degradation occurs in mesophyll cells. No elevation in sugars, or a slight increase in sucrose and/or hyperaccumulation of maltose, has also been observed in these mutants (see Zeeman et al. [2010] for review). Any inhibition in export of photoassimilate from leaves can induce starch excess in the leaves (Additional file 1: Table S1, and references therein). In such LSE mutants in *A. thaliana* and maize, excess starch and sucrose tend to accumulate in leaves. Similarly, we found in rice *lse2* and *lse3* that both starch and sucrose levels in mature leaf blades of seedlings were significantly elevated in comparison with WT (Figure 4). We also found, in an HPLC analysis of carbohydrate contents, that none of *lse1*, *lse2*, *lse3*, or WT contained maltose at detectable levels in leaf blades (Additional file 2: Figure S1). This indicates that neither *lse2* nor *lse3* appear to hyperaccumulate maltose, which was found in a few LSE mutants of *A. thaliana* lacking genes (e.g., *DPE2*) for starch-degrading enzymes (Chia et al. [2004]; Lu and Sharkey [2004]) or for the chloroplast maltose transporter (e.g., *MEX1*; Niittylä et al. [2004]). Thus, the characteristic carbohydrate profiles of rice *lse2* and *lse3* suggest that photoassimilate export, rather than starch degradation, is inhibited. In fact, no elevation in sucrose levels was observed in the leaves of *lse1* seedlings (Figure 4), which lack a starch-degrading enzyme, GWD1 (Hirose et al. [2013]).

As described above, the phloem-localized OsSUT1 does not play an essential role in the phloem-loading mechanism in rice leaves, but the mesophyll (tonoplast)-localized OsSUT2 does play an important role. Since there are five members of the *OsSUT* gene family (Aoki et al. [2003]), it is possible that the other three SUTs (OsSUT3, 4, and/or 5) are involved in apoplastic loading of sucrose in the phloem. However, we found, through an extensive expression analysis of *OsSUT* genes, that the transcripts for *OsSUT3* and *OsSUT5* were extremely low or undetectable in mature leaf blades of rice and that the transcript levels of *OsSUT4* were much lower than those of *OsSUT1* and did not differ between mesophyll tissues and vascular bundle (Additional file 3: Figure S2; also see Aoki et al. [2003]). These results, together with the findings for OsSUT1 and OsSUT2, imply that rice phloem is not loaded by a SUT so a different loading mechanism (e.g., symplastic loading) may be working in source leaf blades. Since either the ossut1 or ossut2 mutant has been reported not to exhibit LSE phenotype (Eom et al. [2011]), we expect that *LSE2* and *LSE3* may be genes that differ from any *OsSUT* s and function in the yet-to-be-elucidated phloem loading mechanism in rice leaves.

In relation to the phloem loading mechanism in rice leaves, the concept of revised diffusion, proposed by Eom et al. ([2012]), is intriguing. This hypothesis is substantially identical to that suggested by Rennie and Turgeon ([2009]). In both reports, the authors hypothesized that passive diffusion of sucrose from the mesophyll to the phloem is accompanied by active pumping of sucrose from the vacuole into the cytosol of the mesophyll cells to maintain higher concentrations of sucrose in the cytosol. An important prerequisite for this hypothesis is that the symplastic connection between the phloem companion cell and the surrounding cells is adequate to allow diffusion of sucrose into the phloem. This requirement appears to be satisfied in rice, according to vein anatomy reported by Kaneko et al. ([1980]) and fluorescent dye movement observed by Scofield et al. ([2007]). Given that the symplastic pathway is the major route of phloem loading of sucrose in rice leaf blades, inhibition of sucrose export in the leaves of *lse2* and *lse3* might be caused by loss of function in the plasmodesmata. In well-characterized maize *sed1*/*sxd1* mutant, export of photoassimilate from mature leaves appears to be inhibited by structural abnormalities in plasmodesmata connecting bundle sheath cells and vascular parenchyma cells, although the involvement of SED1/SXD1 (tocopherol cyclase) in plasmodesmata remains unclear (Russin et al. [1996]; Botha et al. [2000]; Provencher et al. [2001]; Sattler et al. [2003]). We have not observed structural abnormalities in plasmodesmata in leaf blades of *lse2* or *lse3* seedlings under TEM (data not shown). It would first be necessary to identify and characterize the responsible gene, including an in-situ expression analysis, prior to further investigation of the ultrastructure of cells around and within vascular bundles.

The severely impaired growth phenotypes of *lse2* plants (Figures 1 and 3) indicate that the biochemical function of LSE2 is very likely to play a pivotal role in photoassimilate partitioning in rice plants. In transverse sections of *lse2* leaves, hyperaccumulation of starch was observed in mesophyll and bundle sheath cells (Figure 2C,G), suggesting that inhibition may occur in the bundle sheath cells. It is possible that the photoassimilate (sucrose) cannot move into the inner cells of vascular bundles but that excess sucrose can be converted into starch within the bundle sheath cells. This hypothesis is supported by the fact that bundle sheath cells of rice leaf blades can synthesize starch from excess photoassimilate and can also probably degrade it, because hyperaccumulation of starch granules can be found in bundle sheath cells of *lse1* leaves lacking a starch-degrading enzyme (Figure 2B,F). In addition to leaf blades, leaf sheaths of *lse2* seedlings appear to accumulate large amounts of starch and sucrose (Figure 4C,D), suggesting a role for LSE2 in carbohydrate partitioning in leaf sheaths as well. LSE2 is likely to be involved in the remobilization of temporally stored NSCs, presumably functioning in the transport or metabolism of starch-derived sugars, although the apparent hyperaccumulation of NSCs in leaf sheaths may result from the very small volume of tissue in the dwarf plants (Figures 1 and 4). Meanwhile, it is intriguing that the starch-excess phenotype was evident in leaf sheaths of the fifth leaves of *lse2* seedlings (Figure 4C,D) but not in the slowly developing leaf blade (Figure 1; Figure 4A,B). We cannot rule out the possibility that photoassimilates may be transported from the fourth (mature) leaves to the fifth (immature) leaf sheaths, leading to an excess of NSCs that triggers inhibition of photoassimilate partitioning and/or leaf tissue development in the fifth leaf blades.

In *lse3* leaves, on the other hand, starch hyperaccumulation was observed not only in mesophyll and bundle sheath cells, but also within vascular tissues (Figure 2D,H), suggesting the role of LSE3 in photoassimilate transport for phloem loading; movement of sucrose from vascular parenchyma cells to the sieve element/companion cell complex may be inhibited in this mutant. This hypothesis could explain the elevated sucrose levels in leaf blades of *lse3*. However, the extent of inhibition of photoassimilate transport does not appear to be critical, as the impairment of growth in *lse3* plants was not as severe as that observed in *lse2* plants (Figures 1 and 3) and the elevated leaf sugar levels in *lse3* were not as remarkable as those in *lse2*. Interestingly, unlike leaf blades, leaf sheaths and internodes did not exhibit excess accumulation of starch or soluble sugars in *lse3* (Figure 4C,D; Figure 5C–F), in contrast to *lse2* plants. In addition, there was no or only a slight reduction in the percentage of grain filling and grain weight in *lse3* compared with *lse1* and WT plants (Table 2), indicating that LSE3 is unlikely to play a major role in photoassimilate transport and partitioning in filling grains and sink tissues. These results suggest that the physiological importance of LSE3 is limited in the phloem loading pathway of photoassimilates in source leaves.

## Conclusions

The results of phenotypic analysis suggest that LSE2 and LSE3 mutations are caused by the disruption of genes involved in the path of sucrose transport from mesophyll cells to phloem sieve elements in rice leaves. Meanwhile we cannot exclude the possibility that for either *lse2* or *lse3*, the responsible gene is involved in other processes that can indirectly affect photoassimilate partitioning, such as cell and organelle development (Provencher et al. [2001]) or sugar sensing and signaling (Ruan [2014]). Further studies are necessary to characterize the biochemical and physiological functions of LSE2 and LSE3, by identifying *LSE2* and *LSE3* and by expression and localization analysis of these genes in different leaf tissues.

## Methods

### Plant growth and sampling

We previously reported screening of the *Tos17* retrotransposon-insertion mutant collection for rice cultivar ‘Nipponbare’ (Miyao et al. [2003]; https://tos.nias.affrc.go.jp/) by iodine staining to detect starch accumulation in leaves of seedlings (stain-positive plants); as a result, five candidate lines were selected from more than 6300 lines of the M_2_ generation (Hirose et al. [2013]). In addition to a previously established LSE1 mutant line, we selected two other independent lines, which exhibited distinct starch accumulation in leaf blades of 4-week-old seedlings, in this study. During the original screening procedure using the M_2_ generations, both the two lines segregated into stain-positive and stain-negative plants. To establish homozygously mutated lines, the progeny of stain-positive M_2_ plants were examined to check whether all individuals showed the stain-positive phenotype. On the basis of the results of this analysis, we selected the progeny of putative homozygous mutant plants to be the pure line for the mutation, designated the mutations as LSE2 and LSE3, and used these lines for further phenotypic analyses.

Seeds were grown in a plastic tray (30 × 3 × 3 cm) filled with nursery soil in a glasshouse (day/night cycle of 12/12 h, 25/20°C; 60% RH) under natural light with supplementary lighting (350 μmol m^−2^ s^−1^). For starch staining, the aboveground parts of seedlings were boiled in 80% (v/v) ethanol to remove pigments and stained with iodine solution. For the determination of NSCs, plants at the fifth-leaf stage were sampled at the end of the light or dark period, immediately frozen in liquid nitrogen, and stored at –80°C until use.

The LSE mutant and WT (‘Nipponbare’) plants were also grown in paddy fields in 2012 and 2013, at the Institute for Sustainable Agro-ecosystem Services (ISAS), Tokyo, Japan (35°44′N, 139°32′E). Seedlings were grown in a greenhouse for one month and transplanted into the paddy field in late May. The planting density was 22.2 hills m^−2^ (hill spacing of 30 × 15 cm) with one seedling per hill, and compound fertilizer for paddy fields (N:P_2_O_5_:K_2_O = 12:16:18%) was applied at the rate of 50 g m^−2^ as a basal dressing. At the heading stage, following measurement of plant length, the upper tissues of WT, *lse1*, and *lse3* were sampled for measurement of NSCs, immediately frozen in dry ice, and stored at –80°C until use. Plants were harvested in late September, approximately 45 d after heading, and their panicles were used for the analysis of yield components as described in Hirose et al. ([2013]).

### Determination of non-structural carbohydrates

Frozen tissue samples harvested from seedlings were ground using a mortar and pestle under cryogenic conditions and transferred into a pre-weighted 2-mL microfuge tubes for measurement of fresh weight. For field-grown plants, frozen tissues were processed to a powder using a Multi-beads Shocker (MB901U, Yasui Kikai, Osaka, Japan) and transferred into pre-weighted 2-mL microfuge tubes for measurement of fresh weight. NSCs (i.e., starch, sucrose, glucose, and fructose) were extracted and measured as described previously (Hirose et al. [2013]).

### Microscopic observations

Fully expanded leaf blades of 4-week-old seedlings were collected in the morning. For each line, three seedlings were selected and the middle parts of the leaf blades were cut into small segments and used immediately for microscopic observation. The extent of starch accumulation was determined by iodine staining of the rest (tips and bottom parts) of the sampled leaves. The small segments of leaf blades were fixed in 4% (w/v) paraformaldehyde and 2% (w/v) glutaraldehyde in 50 mM phosphate buffer (pH 7.2) for 24 h and post-fixed with 2% (w/v) osmium tetroxide aqueous solution in the same buffer for 2 h at 4°C. Dehydration and resin infiltration were performed in a graded series of acetone solution and propylene oxide. The samples were embedded in Spurr’s epoxy resin (Sigma-Aldrich, St. Louis, MO, USA). Ultrathin transverse sections were prepared with an ultramicrotome (Ultracut UTC, Leica, Germany) and stained with uranyl acetate and lead citrate, and observed using a transmission electron microscope (JEM-1010, JEOL Ltd., Tokyo, Japan) operated at 100 kV. Semithin transverse sections (1 μm thickness) were subjected to periodic acid–Schiff (PAS) staining and observed using a light microscope (Eclipse Ti-S, Nikon, Tokyo, Japan).

## Additional files

## Electronic supplementary material

Additional file 1: Table S1.: Leaf Starch Excess (LSE) mutants in angiosperms. (XLSX 19 KB)

Additional file 2: Figure S1.: Elution profiles of high performance anion-exchange chromatography (HPAEC) for analysis of soluble sugar contents in leaf blades. (PDF 157 KB)

Additional file 3: Figure S2.: Real-time quantitative reverse-transcription PCR analysis of *OsSUT* genes in leaf blades of rice. (PDF 68 KB)

Below are the links to the authors’ original submitted files for images.

Authors’ original file for figure 1

Authors’ original file for figure 2

Authors’ original file for figure 3

Authors’ original file for figure 4

Authors’ original file for figure 5

## Abbreviations

Glc: Glucose
Fru: Fructose
LSE: Leaf starch excess
NSC: Non-structural carbohydrate
PAS: Periodic acid–Schiff
Suc: Sucrose
SUT: Sucrose transporter
WT: Wild type
