# Supplementary material for: Phenotypic analyses of rice lse2 and lse3 mutants that exhibit hyperaccumulation of starch in the leaf blades
Source: Rice (N Y). 2014 Dec 21;7:32. doi: 10.1186/s12284-014-0032-3 (PMC4884028; doi:10.1186/s12284-014-0032-3)
Supplement: Supplementary file 2 — Additional file 2: Figure S1.: Elution profiles of high performance anion-exchange chromatography (HPAEC) for analysis of soluble sugar contents in leaf blades. (PDF 157 KB) [file 12284_2014_32_MOESM2_ESM.pdf]

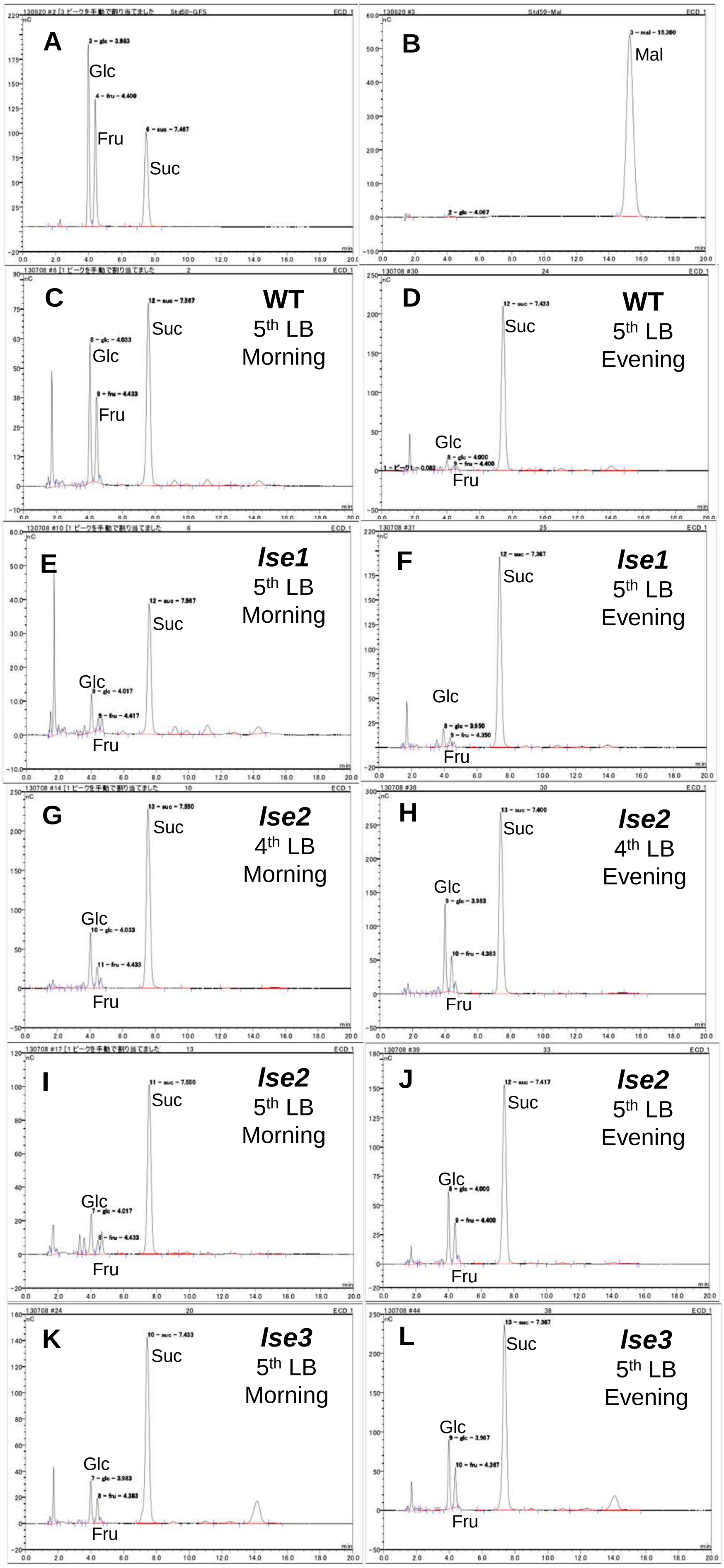

Figure S1. Elution profiles of high performance anion-exchange chromatography (HPAEC) for analysis of soluble sugar contents in leaf blades. Soluble sugar fractions of leaf-blade (LB) samples, used for the determination of non-structural carbohydrate (NSC) levels as shown in Fig. 4, were also subjected to HPAEC analysis using a Dionex DX500 chromatography system (Sunnyvale, CA). Aliquots (20 µL) were separated on a CarboPac PA-1 column with 150 mM sodium hydroxide as the elution buffer (1.0 mL min^{-1}), and integrated pulsed amperometric detection (PAD) was used for carbohydrates with the manufacturer's recommended waveform and Dionex Chromeleon 6.5 software. Peak identification was based on standards (A, B) including sucrose (Suc), glucose (Glc), fructose (Fru), and maltose (Mal). Note that Mal contents appear to be below the detectable level in wild type (WT) (C, D) and *lse* leaves (E–L). It has been reported that Mal levels in leaves of *dpe2* and *mex1* mutants of *A. thaliana* were as high as or even higher than Suc/Glc/Fru levels, while Mal was often observed at the detection limit of HPAEC-PAD in WT plants [Chia et al. (2004) *The Plant Journal* 37, 853-863; Lu and Sharkey (2004) *Planta* 218, 466-473; Niittylä et al. (2004) *Science* 303, 87-89]. Therefore, the rice *lse* mutants do not appear to exhibit hyperaccumulation of Mal in leaves.
