# Supplementary material for: Phenotypic analyses of rice lse2 and lse3 mutants that exhibit hyperaccumulation of starch in the leaf blades
Source: Rice (N Y). 2014 Dec 21;7:32. doi: 10.1186/s12284-014-0032-3 (PMC4884028; doi:10.1186/s12284-014-0032-3)
Supplement: Supplementary file 3 — Additional file 3: Figure S2.: Real-time quantitative reverse-transcription PCR analysis of OsSUT genes in leaf blades of rice. (PDF 68 KB) [file 12284_2014_32_MOESM3_ESM.pdf]

A

Gene	Locus ID*	Left primer (5' to 3')	Right primer (5' to 3')
OsSUT1	Os03g0170900	tcatccctcaggtggtcatcg	cttggagatcttgggcagcag
OsSUT2	Os12g0641400	gtcataccacaggttattgtgtc	gaattgcaaagaatggccg
OsSUT3	Os10g0404500	tcctcttcgacaccgactg	cagcacgatcgagttaaggag
OsSUT4	Os02g0827200	cgttgttccgcagatagtagtg	gtgttctgctcagccaaatcc
OsSUT5	Os02g0576600	tcggcatggtgtccatgag	caatggcaagaccttggcc
RUBIQ1	Os06g0681400	ggagctgctgctgttcttgg	cacaatgaaacgggacacga

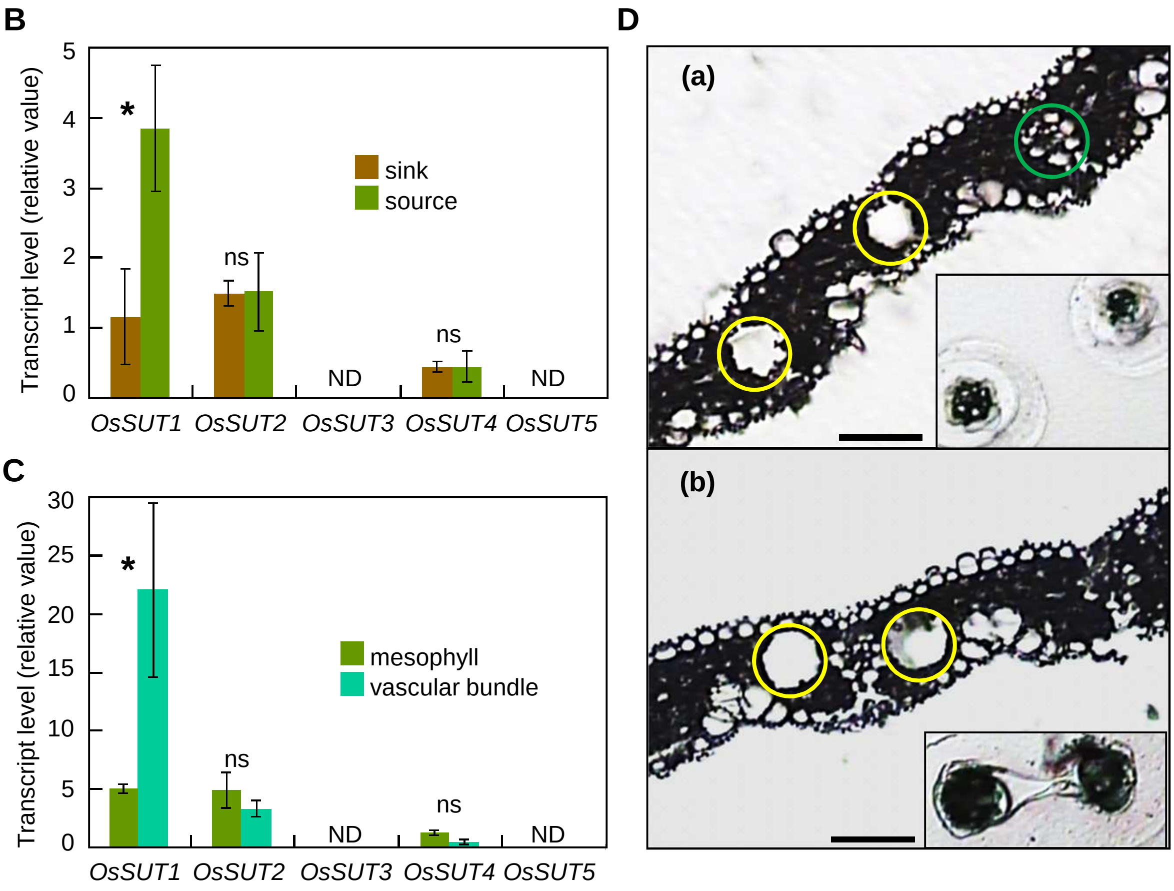

Figure S2. Real-time quantitative reverse-transcription PCR (Q-PCR) analysis of *OsSUT* genes in leaf blades (LBs) of rice. (A) The nucleotide sequences of the primers used in the Q-PCR analysis. Locus IDs were based on the RAP_DB (http://rapdb.dna.affrc.go.jp/). (B) Comparison of transcript levels of five *OsSUT* genes between sink and source LBs. Seedlings (cv. Nipponbare) were grown until the seventh leaf was fully elongated. The LBs of the 7th leaf were then sampled as source LBs. The elongating eighth leaf, which was covered by the sheath of the seventh leaf, was sampled as the sink LB. RNA was extracted from LB samples using an RNeasy Plant Mini kit (Qiagen), reverse-transcribed with Superscript II reverse-transcriptase (Life Technologies), and used as templates for Q-PCR with SYBR Premix ExTaq II (Takara Bio) and Smart Cycler II (Cepheid). The results obtained for the *OsSUT* transcripts were normalized to the transcript level of a rice polyubiquitin gene [*RUIBQ1*; Wang et al. (2000) *Plant Science* 156, 201-211], and expressed as relative values. Data are presented as the means ± SD. ND, not detected. (C) Comparison of transcript levels of five *OsSUT* genes between mesophyll and vascular bundles of source LBs. The LBs of fully elongated seventh leaves were sampled from rice seedlings grown as described above, fixed with ice-cold acetone, embedded in paraffin (Paraplast-XTra, Sigma), and used for laser microdissection as described previously [Hirose et al. (2010) *Journal of Experimental Botany* 61, 3639-3646]. The small vascular bundles and mesophyll tissue were collected separately, and total RNA was extracted using a PicoPure RNA isolation kit (Arcturus). Approximately 100 pg/reaction of total RNA was subjected to Q-PCR using a One Step SYBR PrimeScript RT-PCR Kit II (Takara Bio Inc.), carried out as described above. (D) Typical images showing the procedure of laser microdissection. Cross sections of source LBs are shown. Vascular bundles (a) or mesophyll tissues (b) were cut off as indicated by yellow circles, and isolated (inserts). Green circle indicates the vascular bundle that was not cut off. Scale bars = 0.1 mm.
