# Supplementary figures and images for: Phenotypic analyses of rice lse2 and lse3 mutants that exhibit hyperaccumulation of starch in the leaf blades
Source: Rice (N Y). 2014 Dec 21;7:32. doi: 10.1186/s12284-014-0032-3 (PMC4884028; doi:10.1186/s12284-014-0032-3)

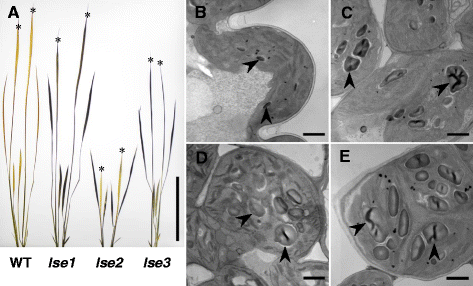

Supplement: Supplementary file 4 — Authors’ original file for figure 1 [file 12284_2014_32_MOESM4_ESM.gif]

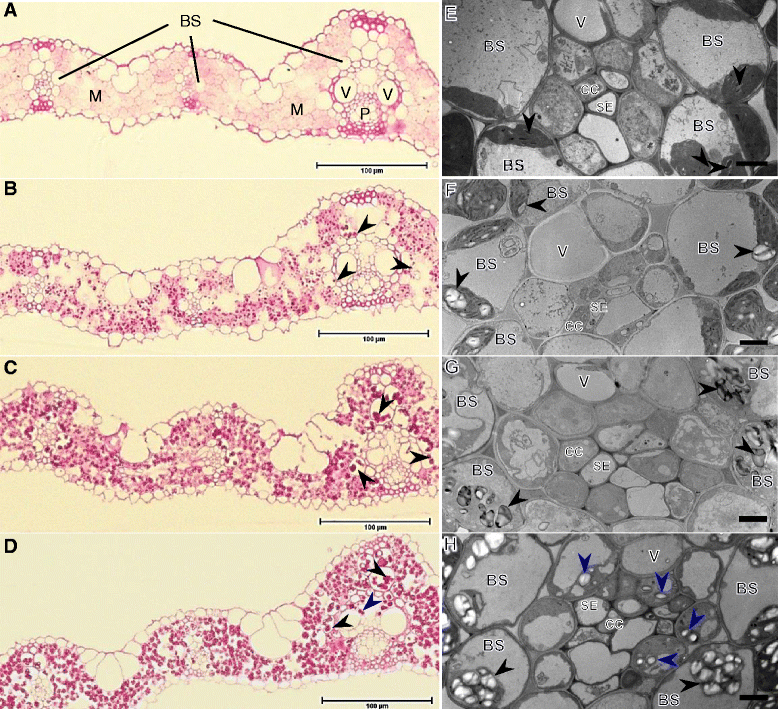

Supplement: Supplementary file 5 — Authors’ original file for figure 2 [file 12284_2014_32_MOESM5_ESM.gif]

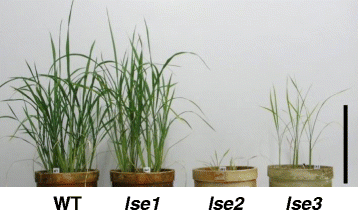

Supplement: Supplementary file 6 — Authors’ original file for figure 3 [file 12284_2014_32_MOESM6_ESM.gif]

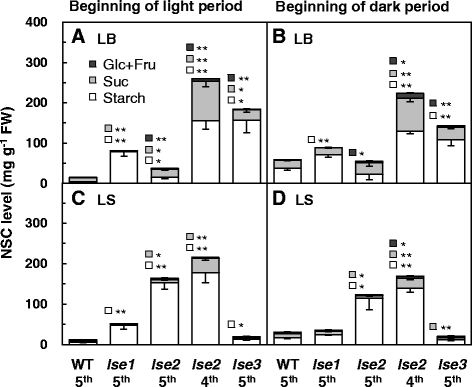

Supplement: Supplementary file 7 — Authors’ original file for figure 4 [file 12284_2014_32_MOESM7_ESM.gif]

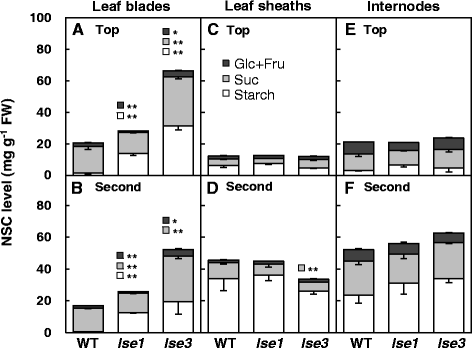

Supplement: Supplementary file 8 — Authors’ original file for figure 5 [file 12284_2014_32_MOESM8_ESM.gif]
